# Freedom of the General Nursing Council Imperilled

**Published:** 1921-05-07

**Authors:** 


### Freedom of the General Nursing Council Imperilled.

The long-continued delay in the issue of the rules for registration by the General Nursing Council for England and Wales is now authoritatively stated by the Minister of Health to be due to the framing by the Council of a rule "purporting to give them a discretion to refuse to admit to their Register nurses already on the Scottish and Irish Registers." The lawyers appear to have decided that the words in the Nurses' Registration Act which relate that "with a view to securing a uniform standard of qualification in all parts of the United Kingdom, the Council shall, before making any rules under this Act with respect to the conditions of admission to the Register, consult with any Nursing Councils which may be established by Parliament for Scotland and Ireland respectively," debar the English Council from its discretionary powers as regards the admission of existing nurses. In short, we are face to face with a decision on the part of the Ministry of Health calculated to compel the General Nursing Council for England willy-nilly to admit to the English Register whatever nurses it may be decided to register in Scotland and Ireland. If this decision be adhered to, something like a deadlock has occurred. For the Council was given definite powers only to admit *bonâ fide* nurses to the Register who have "adequate knowledge and experience of the nursing of the sick," and it is surely their business to decide what that implies.

It will be a great misfortune for the English Register if the Council is pinned down to accept a standard of which it disapproves. After all, the Act only says the English Council must "consult with" the other Councils. It does not say it must succeed in coming to an agreement with them. And when the Irish Council agreed to register "qualified nurses," we fear it rendered the chances of agreement very slender. It is much to be regretted that the three Councils did not come to a decision on this matter before the issue by the Irish Council of its rules.

